# Academic Stress and Mental Health Challenges Among International Students in China

**DOI:** 10.3390/bs16071212

**Published:** 2026-07-17

**Authors:** Dongxia Zeng, Asim Zubair, Haijun Dong

**Affiliations:** 1 School of Law and Public Administration, Hunan University of Science and Technology, Xiangtan 411199, China; 1190017@hnust.edu.cn; 2 School of Public Administration, Hohai University, Nanjing 210098, China; 3 School of Public Administration, Central South University, Changsha 410083, China; donghj34@csu.edu.cn

**Keywords:** academic stress, mental health, international students, higher education, China

## Abstract

International students in China frequently encounter academic and acculturative challenges that adversely affect their psychological wellbeing. However, limited research has integrated quantitative and qualitative evidence to comprehensively examine the determinants of stress, anxiety, and depression among this population. This study investigated the effects of academic and acculturative stressors on the mental health of international students and explored their coping experiences and perceptions of institutional support. An explanatory sequential mixed-methods technique was employed. Quantitative data were collected from 500 international students enrolled at four universities in Changsha, China, followed by semi structured interviews with 10 participants. Confirmatory factor analysis demonstrated satisfactory construct validity (CFI = 0.93, TLI = 0.92, RMSEA = 0.052, SRMR = 0.047). Hierarchical regression and thematic analysis were used to analyze the quantitative and qualitative data, respectively. Academic workload emerged as the strongest predictor of psychological distress, significantly predicting stress (β = 0.37, *p* < 0.001), anxiety (β = 0.34, *p* = 0.001), and depression (β = 0.31, *p* = 0.003). Language barriers and social isolation were also significantly associated with poorer mental health outcomes. Qualitative findings further revealed that research and publication pressures, cultural adjustment difficulties, and inadequate institutional support intensified students’ psychological distress, whereas academic mentoring and peer support enhanced coping and resilience. Strengthening culturally responsive counseling, language support, academic mentoring, and social integration programs can substantially improve international students’ mental wellbeing and academic success. These findings provide practical evidence for developing inclusive university support policies in China.

## 1. Introduction

International students studying in China have increased rapidly in the past decades, with over 492,000 enrolled in 2018. For all students, having studied abroad can be such a memorable experience; however, it can also be very challenging when moving into a new cultural and academic environment. These challenges, both physical and psychological, have led to an increase in international students experiencing mental health problems. Studies have shown that mental health problems are a global concern within higher education and that academic stress is the main contributing factor to burnout in students. Evidence from a large epidemiological study of students across 18 countries demonstrates that one in three students has a mental health disorder (Qian & Yu, 2023). Student mental health has thus become the top priority for colleges and universities around the world (Gong et al., 2021).

In the academic setting, numerous studies have confirmed the detrimental effects of academic stress. More specifically, academic stress has been associated with higher levels of anxiety, depression and burnout (Sun et al., 2012; Hussain & Shen, 2019), which have been shown to impair academic achievement. Results also suggested that academic demands may also have long-term effects on both psychological wellbeing and academic engagement as the academic year progresses (B. Yu et al., 2014).

International students in China are particularly at risk of experiencing adverse effects of acculturative stress as they may be faced with the compounding effect of multiple aspects of cultural adjustment and academic pressures (Li, 2015; Ma & Zhao, 2018). Acculturation refers to a process of cultural adaptation that occurs when an individual or group is exposed to a different culture following a major life event. In accordance with the model of acculturative stress proposed by Berry (2006), it is thought that the adaptation to a new culture and being subjected to the perception of discrimination or shortages of friends would cause accessibility of a multitude of negative mental health outcomes.

The Chinese higher education context presents a series of challenges for international students, including heightened competition for entrance focuses, hierarchical teacher–student relationships, the importance of examination performance, and a collective approach to academic achievement (Ma & Zhao, 2018). These factors may be compounded by the language difficulties, cultural adjustment, and academic pressure that international students face, increasing stress and impeding psychological wellbeing, academic performance, and retention (Yang & de Wit, 2019; Zhu et al., 2022).

The cultural stigma surrounding mental health in China also makes students reluctant to seek help. Collectivist values centered on family honor (i.e., mianzi) selectively reinforce distress being internalized among students, especially males, who are reluctant to seek formal professional help with mental health and are aligning themselves through informal networks in order not to socially expose themselves or burden others (S. Yu et al., 2018; Xu et al., 2018; Cao et al., 2023). This highlights the importance of developing culturally sensitive interventions and support systems that deal with academic–acculturative stress and mental wellbeing among international students (An & Chiang, 2015; Li, 2015; Ding, 2016; Jiang et al., 2018; Wang et al., 2023).

The interconnections of academic stress, acculturative issues and psychological distress points to the need for inclusive effective strategies including those that bolster mental health among international students in support of globalized higher education initiatives in China.

### 1.1. Research Gap

Despite the rapid increase in international students studying at Chinese universities, research on academic stress and mental health among international students in China is very limited. Most previous research has only concerned cultural adaption and the language barrier, leaving aside the effect of China’s academic environment on international students’ psychological health.

Some aspects of Confucianism, such as discipline, being proactive, struggling to obtain the best results in exams, and serving and respecting elders and authority (e.g., teachers, parents, manager), are all spread deeply into Chinese higher education. The adherence to such academic norms without balances, combined with the impacts of the diverse students return and educational backgrounds (e.g., Western, European and African) could increase the stress of international students, affecting their academic motivation, and worsening their mental health problems.

However, this shortage is still an important aspect of studies that only focus on the association of cultural adaptation, academic stress and mental health problems among international students in Chinese higher education. The purpose of this research is to evaluate the association between academic stress and students’ mental health, and further, to look into how Chinese institutions could provide inclusive institutional & psychological support for diverse international students.

### 1.2. Theoretical Framework

**Figure 1:**
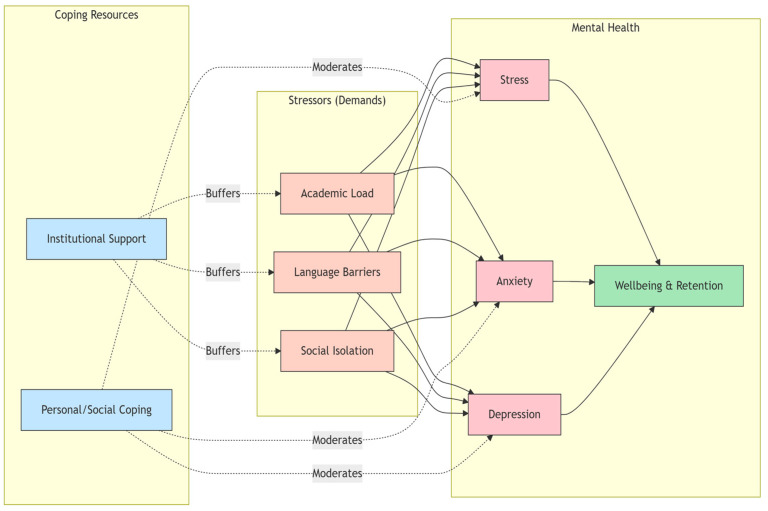
Theoretical framework of academic stress and mental health among international students in China.

The present study is grounded in a theoretically rich overview of how stress can affect international students’ mental health. The JD-R theory (Bakker & Demerouti, 2017) suggests that the way in which individuals perceive and respond to demands depends on resources support available to them. Scott’s Transactional Model of Stress and Coping proposes how individuals appraise and cope with demands and stress. The framework is situated within acculturative stress theory (Berry, 2006) to understand how cultural adaptation challenges including language barriers, social isolation, and unfamiliar academic norms affect international students’ psychological health in China. This perspective may expand on previous research by suggesting that academic demands and challenges in cultural adjustment are major stressors that negatively impact students’ mental health. At the same time, institutional support, social integration, and coping strategies can mitigate these potential harms. The framework further suggests that the interaction of academic and cultural demands may trigger stress, aggression, and emotional distress, and may threaten the retention and success of international students. Based on this perspective, this study aims to investigate the relationship between academic stress and mental health challenges faced by international students in China, with implications for identifying factors that support student resilience and wellbeing. See Figure 1.

## 2. Research Methodology

### 2.1. Research Design

This study employed a cross-sectional mixed-methods research design. Quantitative data were collected through a structured questionnaire administered to 500 international students, while qualitative data were obtained through semi-structured interviews with 10 purposively selected participants to gain a deeper understanding of their experiences of academic stress and mental health challenges in China. The integration of quantitative and qualitative approaches within a single research project has been widely recognized as an effective methodological strategy in the social sciences (Creswell, 1999). Specifically, this study adopted an explanatory sequential mixed-methods design, in which quantitative data were collected and analyzed during the first phase, followed by qualitative interviews to further explain and enrich the quantitative findings. This approach enabled a more comprehensive understanding of how academic and acculturative stress influence the mental health of international students in China (Ivankova et al., 2006).

### 2.2. Study Area and Sample Size

The study was carried out in Changsha, China, at Central South University, Hunan University, Hunan Normal University and Changsha University of Science and Technology, which have a wide variety of international students. In the quantitative phase, 500 international students studying in undergraduate, master’s and PhD programs were recruited for this study. A total of 650 questionnaires were distributed, with 500 returned (response rate of 76.9%). Follow-up visits to increase participation were conducted, although no formal nonresponse analysis was performed. The qualitative phase purposively sampled 10 respondents from the survey to provide maximum variability in terms of known demographics: degree level, gender and country; and self-reported stress scores (low, moderate, high). Data were collected from August 2025 to March 2026, depending on participant availability.

### 2.3. Sampling Method

The quantitative phase employed a stratified random sampling approach to guarantee the proportional representation of degree levels and participating universities. The sampling frame comprised the official lists of all international students enrolled at Central South University, Hunan University, Hunan Normal University and Changsha University of Science and Technology for the 2025/2026 academic year. The initial stratification in the population was taken by university and then for each degree level (undergraduate, master’s, and PhD). A quantitative structured questionnaire was offered to international students who experience academic stress and mental health challenges. Purposive sampling was used for the qualitative phase, where 10 of the respondents from survey were selected based on variations in stress levels, type of degree program, gender and country of origin. Semi-structured in-depth interviews were carried out to elicit rich accounts of the impact of academic adjustment, cultural adaptation, coping behaviors, and mental health problems faced by Chinese higher education students.

### 2.4. Data Collection

Method Quantitative phase: The questionnaire was composed of measures with established reliability and validity. Perceived academic stress, language barriers and social isolation were assessed through 5-point Likert scale items, with endpoints “every day” and “never”. Institutional support was also assessed on a 5-point scale (1 = low support, 5 = high support). Mental health problems were assessed through 4 items regarding the occurrence of stress, anxiety, and depressive symptomatology (“Not at all”, “Only a little”, “Quite a lot”, “A lot”). Responses of “Quite a lot” and “A lot” were labeled as medium to high levels of stress, anxiety and depression. Demographic variables included gender, age at time of study group, region of residence and educational level. Qualitative phase participants were used in this stage to conduct semi structured in depth interviews with 10 participants on four topics: (a) academic stress and mental health, (b) coping strategies, (c) institutional support and (d) cultural adjustment. Each interview lasted an average of 30–40 min, was audio-taped (with permission n = 10), and transcribed verbatim for data analysis. Interviews were conducted in English or in the participant’s language of preference.

All key variables in the study (academic load, language barriers, social isolation, and institutional support) were measured using structured multi-item scales on a 5-point Likert scale (1 = strongly disagree to 5 = strongly agree). Academic load items focused on workload, assignment pressure, and academic research demands. The remaining constructs assessed perceived communication difficulties, social isolation and lack of integration, and the quality of institutional support. Mental health outcomes (stress, anxiety, and depression) were measured using brief self-report screening items adapted from established higher education instruments. Responses were recorded on a 4-point frequency scale (“not at all” to “a lot”) over the past month, and then recoded for analysis, with “quite a lot” and “a lot” coded as 1 (moderate-to-high symptoms) and “not at all” and “only a little” coded as 0 (low symptoms), consistent with prior epidemiological screening approaches.

For regression analyses, mental health outcomes (stress, anxiety, and depression) were treated as continuous variables by summing item scores to create composite scale scores. This approach is consistent with established practices in mental health research where ordinal response items are aggregated to form continuous measures of symptom severity. The internal consistency of these composite measures was acceptable (Cronbach’s α = 0.74–0.84). While the individual response categories were ordinal, the summed scores were treated as approximately continuous, justifying the use of linear regression.

Although these items do not constitute full diagnostic scales, they only constitute mild to moderate levels of global distress, and thus would unlikely lead to diagnostic misclassification. Nevertheless, a confirmatory factor analysis (CFA) revealed that the six items loaded well onto five single-factor constructs (loadings ranged from 0.51 to 0.78). The reliabilities for the items were good and ranged from 0.74 to 0.84 across the various constructs. These results indicate that the six shortened items captured well the major dimensions of distress.

### 2.5. Data Analysis

Quantitative data were analyzed using SPSS 27.0 and AMOS 27.0. Frequencies, percentages, means and standard deviations were used to describe participants’ demographic characteristics and study variables. The hypothesis testing the validity of the measurement model was established through Confirmatory Factor Analysis (CFA) prior to hypothesis testing. Model fit was assessed with the Comparative Fit Index (CFI), Tucker–Lewis Index (TLI), Root Mean Square Error of Approximation (RMSEA) and Standardized Root Mean Residual (SRMR). Descriptive and inferential statistics and Pearson’s correlation analysis were used to analyze relationship between academic stress, mental health challenges, language barriers, social isolation and institutional support. A subsequent hierarchical linear regression analysis was performed to determine significant predictors of mental health problems among international students. Statistical significance was set at *p* < 0.05. The CFA model fit indices are presented in Table 1.

Prior to hierarchical regression, all assumptions of linear regression were tested. Linearity was checked using residual and partial regression plots, while normality of residuals was assessed via Shapiro–Wilk test and Q–Q plots. Homoscedasticity was examined through standardized residuals versus predicted values. Multicollinearity was evaluated using VIF and tolerance (VIF < 5 considered acceptable). Outliers were identified using Cook’s distance (>1) and standardized residuals (±3). All diagnostics indicated that the assumptions were adequately met.

Qualitative Phase: Qualitative data were analyzed using Braun and Clarke (2006) thematic analysis of interview transcripts. Interviews were transcribed verbatim and read repeatedly to become familiar with the data. Step 1: Up to this step, the research conducted was based on open coding which resulted in a number of initial codes that were grouped under broader themes, namely, academic stress, coping strategies, institutional support and cultural adjustment. The coding decisions and emergent themes were discussed by the research team to increase credibility, as well as consistency. Constant comparative analysis of the data was done alongside data collection until thematic saturation was reached (i.e., the point at which no new themes were emerging from subsequent interviews). Manual coding, theme development, and interpretation of participants experiences comprised all qualitative analyses.

The hierarchical regression model was specified according to the theoretical justification derived from the JD-R framework and transactional model of stress and coping. Demographic variables were entered in step 1 along with academic variables in step 2 and social variables in step 3. This sequence allowed for the assessment of variance explained by the academic and social variables after accounting for demographic variables.

To ensure robustness of the regression results, multicollinearity diagnostics were conducted using Variance Inflation Factor (VIF) and tolerance values. All VIF values were below the recommended threshold of 5, indicating no serious multicollinearity concerns among predictors. Correlation coefficients among independent variables were examined and remained within acceptable ranges, confirming that although predictors were related, they were not statistically redundant.

In addition, alternative model specifications were tested by excluding individual predictor blocks and re-estimating the regression models. The direction and significance of key predictors (academic load, social isolation, and language barriers) remained stable across models, supporting the robustness of the findings.

### 2.6. Ethical Considerations and Informed Consent

The study entitled “Academic Stress and Mental Health Challenges Among International Students in China” was approved by an institutional Ethics Review Committee. Since vulnerable populations and minors were not involved and risks to participants were minimal, the Committee deemed that the study was eligible for exemption. Standard quantitative survey methodology, along with qualitative semi structured interviews, were used in the research on a voluntary basis and anonymously.

Ethical statement: The study was conducted in accordance with the ethical principles of the 1964 Declaration of Helsinki and its later amendments. Participants were fully informed of the purpose, procedures, and voluntary nature of the study before data collection and informed consent was obtained from all participants. The research was carried out in complete anonymity and confidentiality. Participants were assured that no identifiers would be released and that they may exit the study at any time without negative consequences.

## 3. Results

The data shown in Table 2 represents the demographic characteristics of 500 international students surveyed in Changsha, highlighting key patterns across gender, age, region, and academic level. The sample consists of slightly more males (n = 280; 56.0%) than females (n = 220; 44.0%). In terms of age distribution, the largest group falls within the 26–30 years category (n = 197; 39.4%), followed by 31–40 years (n = 169; 33.8%) and 20–25 years (n = 134; 26.8%). Regarding regional background, the majority of respondents are Asian (n = 290; 58.0%), while African students account for 210 (42.0%). Academically, master’s students constitute the largest proportion (n = 225; 45.0%), followed by PhD students (n = 175; 35.0%) and undergraduate students (n = 100; 20.0%). Overall, the findings indicate a predominantly postgraduate and relatively mature student sample, which may be more exposed to heightened academic demands and related stressors. Demographic Characteristics of international students presented in Table 2.

Table 3 also demonstrates a high prevalence of mental health symptoms among the international students. A fairly large number of students reported experiencing stress, anxiety, or some depression at ‘often’ or ‘always’ levels, indicating a relatively high level of psychological distress. The findings from this study suggest that the pressure caused by the intensity of the academic workload, adjustment issues, and acculturative stress are all acting as stressors to cause this mental health problem. In summary, this study underscores the importance of the development of several tailored resource strategies to effectively help these students cope with their mental health problems. Prevalence of mental health symptoms are discussed in Table 3.

### 3.1. Hierarchical Regression Analysis

Table 4 presents findings from hierarchical linear regression, showing the cumulative contribution of demographic, academic and social predictors to variance in stress, anxiety and depression scores. The analysis was carried out in three steps. In general, demographics (Step 1) reveal that female gender is a strong positive predictor for all three outcomes (i.e., β = 0.18–0.22, *p* < 0.05), whilst age has a non-significant negative trend, explaining in total only 1.7–2.1% of variance (R^2^). Second, academic variables (Step 2) explain much more variance (ΔR^2^ = 0.102–0.107), with PhD status (β = 0.24–0.28, *p* < 0.01) and especially higher academic load (β = 0.31–0.37, *p* < 0.001) being consistently strong significant positive predictors independent of the cultural factors listed in step 1. Finally, social factors (step 3) enter in a further substantial increment (ΔR^2^ = 0.024–0.035): both social isolation (β = 0.19–0.25, *p* < 0.05) and language barriers (β = 0.14–0.17, *p* < 0.05) are significant positive predictors, leading to total models that explain a total of 15.2–15.7% of the variance in outcomes such as mental health over and above known prognostic factors. Hierarchical regression analyses predicting stress, anxiety, and depression among international students are presented in Table 4.

These quantitative findings are supported by qualitative evidence, with participants describing the consistent academic workload, research pressure, and social isolation that they face as responsibilities that contribute to their psychological distress.

Table 5 presents a joint display that integrates quantitative findings with qualitative themes to provide a more comprehensive understanding of international students’ mental health challenges. The statistical results identify major predictors of stress, anxiety, and depression, while students’ narratives explain how these factors influence their daily academic and social experiences. The qualitative themes support the quantitative findings by highlighting issues such as workload pressure, research burden, social isolation, language difficulties, and barriers to counseling services. Together, these findings provide a deeper interpretation of the factors affecting students’ psychological wellbeing. Quantitative findings and supporting qualitative themes explained in Table 5.

### 3.2. Correlations Among Stressors and Mental Health

As Table 6 shows, the correlation matrix uncovered significant associations between the academic stressors and mental health indicators. Not surprisingly, academic load showed the strongest correlations, with a large effect size to stress (r = 0.58, *p* < 0.01) and anxiety (r = 0.53, *p* < 0.01), which indicated that this single domain of academic stressors may have multidimensional effects on student’s mental health. Moderate but statistically significant relationships between the language barriers and social isolation variables were observed with self-reported stress (r ranging from 0.27 to 0.33, all *p* < 0.01), which indicated that while all of these independent variables led to some portion of negative effects, the effects of academic demand may have been more prominent than the other social factors. A correlation of 0.68 between stress and anxiety was observed, which indicates that these two constructs are highly comorbid. Both of these findings are consistent with previous research and demonstrate the importance of analyzing the simultaneous effects of these stressors on student wellbeing. Correlation matrix of stressors and mental health discussed in Table 6.

Table 7 presents the full measurement model including academic stress (academic load), anxiety, depression, institutional support, language barriers, and social isolation.

### 3.3. Validity and Reliability of the Data

To assess the overall measurement quality, measurement validity, and reliability, the four scales adopted in this study, namely student stress, international student adjustment, perceived institutional support, and brief mental health screening tools, were first examined for validity and reliability. CFA results reported in tables showed that all factor loadings were above the acceptable threshold of 0.50 (Hair et al., 2019). In addition, all estimated composite reliabilities (CRs) were above 0.60 (Bagozzi & Yi, 1989), with the lowest value at 0.66, signaling acceptable construct reliability. Although some AVE results of those items were at the borderline of 0.40, all AVE results of the scales met the acceptable threshold of 0.40 (Fornell & Larcker, 1981).

To establish the psychometric robustness of the measurement model, a confirmatory factor analysis (CFA) was conducted for the four latent constructs: academic stress, anxiety, depression, and support services, specified as reflective and allowed to correlate. Model fit was evaluated using multiple indices, including Comparative Fit Index (CFI) and Tucker–Lewis Index (TLI), where values ≥0.90 indicated acceptable fit and ≥0.95 indicated good fit. The Root Mean Square Error of Approximation (RMSEA ≤ 0.08) and Standardized Root Mean Square Residual (SRMR ≤ 0.08) were also used to assess model adequacy. The CFA results indicated an acceptable-to-good model fit for the data (see Table 7). In addition, all standardized factor loadings were statistically significant (*p* < 0.001) and exceeded the recommended threshold of 0.50 (range: 0.51–0.79), confirming convergent validity at the item level.

### 3.4. Stress Distribution by Degree Level

Table 8 shows the distributions of stress levels of the samples. The patterns between this table and Table 7 are very similar among these three groups. The distribution of stress levels of respondents entering master’s programs is very different from the distribution of stress levels of students in other two programs. The second most common level of stress among the master’s students is high stress, as 35.6% of respondents were experiencing high levels of stress, which is more than the 25.0% of respondents experiencing high stress from the undergraduate students, and the 28.6% of respondents experiencing high stress from the PhD students. It is worth noting that 16.4% of the master’s students considered themselves to experience low stress levels, while the PhD student figure was 25.7% and the undergraduate one was only 22.0% respectively. This demonstrates that the master’s students might have a more polarized distribution in terms of their level of stress compared to the distribution of undergraduates and PhD students.

The averages in Table 9 showed high means for every aspect of research and publication pressure that I asked. These results were echoed and supported by the qualitative interview findings, which pointed to publication demand and thesis pressure as key stressors.

### 3.5. Top Academic Stressors

The most frequently reported academic stressors include publication pressure (with 55.0% of the sample reporting) and time to publication as the top stresses. Other top stresses include thesis and dissertation writing and an expectancy to publish (Eisenberg et al., 2007; Levecque et al., 2017). The key finding here is that the most common stressors reported by students in Table 9 are those that may be more difficult to alter compared to the other measures. As the data shows, these stressors are distributed differently by degree. Undergraduates reported the highest proportion of high levels of stress at 25.0% and master’s students the highest proportion of high levels of stress at 35.6%, followed by PhD students with 28.6% very high stress. This data suggests that master’s level students may be at higher risk, even if they do not report the highest confusion or uncertainty stresses.

### 3.6. Institutional Support Utilization and Satisfaction

Table 10 shows that almost two thirds (65.0%) of participants had made use of the thesis/dissertation support workshops, with a mean satisfaction of 4.05 (SD = 0.74). Only slightly more than one third (38.0%) of participants had used the counseling and psychological services, with a mean satisfaction of 3.02 (SD = 1.28). This was dramatically different from academic advising, which was used by 58.0% of participants with a mean satisfaction of 3.71 (SD = 0.68). The 20-percentage-point gap between the 58.0% of participants who used academic advising and the 38.0% who used counseling and psychological services was surprising and may indicate one or both of several factors of inaccessibility of and/or stigma surrounding mental health services. It also indicates the very different experiences of high-stress students with these two types of support services at the university.

### 3.7. Qualitative Insights from in Depth Interviews

For the qualitative phase, semi-structured interviews were performed on 10 international students across different genders, degree levels and countries of origin. We conducted qualitative interviews to explore however participants’ experiences of academic stress and mental health challenges, cultural adjustment, coping strategies manifested while studying in China. All interviews were held in either English or participants’ preferred languages to ensure accurate communication and expression of experiences. Thematic analyses were performed by transcribing and translating the data following empirical procedure. Several interrelated themes emerged from the findings: academic workload and research pressure, language issues, low social interaction or social isolation, supervisor related stress, mental health effects, and coping strategy.

Each of the interviews revealed a central theme: those international students were inundated with coursework. Respondents often characterized a high-pressure cycle of assignments, exams, presentations and research activities that appeared to afford them limited respite or focus on health. It was reported that master’s students and PhD students were particularly under pressure of research expectations for publications. This result is consistent with the quantitative findings, where the most important academic stressors were research/publication burden (M = 4.50) and thesis/dissertation pressure (M = 4.10).

*“When I finish one assignment, another deadline immediately appears. It feels like there is never a break. The workload is continuous, and sometimes I feel mentally exhausted just trying to keep up.”*

(Master’s student, Asia)

Doctoral students were particularly stressed regarding research and publication expectations. As several participants noted, the deliberately created pressure to publish in high-impact journals led to concern and unclear guidelines regarding career progression.

*“My supervisor encouraged me to target a Q1 SSCI journal instead of a journal I originally selected. The expectations became much higher, and the rejection of my manuscript seriously affected my confidence and mental health.”*

(PhD student, Africa)

Language barriers proved to be another major hurdle. While many programs were delivered in English, there were some reports that even coursework was challenging as well, as an inability to understand the technical terminology of courses, discussions during classes, and communication outside the classroom. Often these barriers limited academic participation along with social participation.

*“Language is one of the biggest challenges. Even after studying here for a long time, daily communication can still be difficult. Sometimes I hesitate to ask questions because I am not confident in expressing myself.”*

(Master’s student, Asia)

Interviews also revealed a link between language challenges and school performance. One student noted that the lack of understanding during lectures and discussions increased anxiety and decreased confidence.

*“Some lectures move very fast, and if I miss one concept because of language difficulties, it becomes difficult to follow the rest of the class. That creates a lot of stress before examinations.”*

(Undergraduate student, Africa)

Another common response was that social isolation has been real. In these testimonials, many students described feelings of loneliness, little engagement with local students and struggles in developing lasting friendships. These results reinforced the quantitative findings where social isolation was a significant predictor of stress, anxiety and depression.

*“Back home, studying was a social activity. Here, I often study and eat alone. After several months, I still feel disconnected from people around me, which affects my motivation and emotional wellbeing.”*

(PhD student, South Asia)

A few respondents reported that cultural gaps and limited language capability, at times, led to restrictions learning with peers or interacting with laboratory staff and scientists leading to feelings of exclusion.

*“I tried to communicate with my classmates and lab members, but because of language and cultural differences, I often felt ignored or left out of conversations.”*

(Master’s student, Africa)

Academic stress has been alluded to numerous mental health concerns, e.g., anxiety, sleep disturbances, fatigue and emotional exhaustion. Ensuring an optimal balance between academics and their personal life was referred to as the juggle by participants.

*“There were periods when I could not sleep properly because I kept thinking about research deadlines, presentations, and exams. The stress affected both my concentration and physical health.”*

(PhD student, Asia)

Students noted that logistical issues, such as long commutes and early morning classes, contributed towards fatigue and low psychological wellbeing.

*“I have to wake up very early and travel a long distance to attend classes. The constant disruption of my sleep schedule makes me tired and stressed throughout the semester.”*

(Undergraduate student, Africa)

However, participants reported several coping strategies regarding these difficulties. Academic pressure was something that many students were given the ability to cope with through support from friends/peer networks, exercising, engaging in religious practices and personal resilience. However, barriers to accessing existing mental health services at the university were seen in relation to cultural stigma and the limited awareness and language of university-based support services.

*“Talking with other international students helps because they understand the same struggles. We support each other emotionally, but I think universities need more accessible mental health services specifically designed for international students.”*

(Master’s student, Asia)

In sum, the qualitative results indicate that there are multiple factors related to mental health challenges posed by academic workload, research and publication demands placed on students, language barriers experienced, and social isolation among international students in China. Methods: Although students draw on a range of coping mechanisms, from the interviews, we identify areas for better institutional support that is culturally appropriate. These include aspects of counseling, academic mentoring and social integration best suited to enhancing student wellbeing and academic attainment. Qualitative results are also shown in Table 11.

## 4. Discussion

This study demonstrated a strong convergence between the quantitative and qualitative findings. Quantitative analyses identified academic workload as the most significant predictor of stress, anxiety, and depression, while social isolation and language barriers also emerged as important determinants of psychological distress. These findings were reinforced by the qualitative data, in which participants consistently described heavy academic demands, research and publication requirements, thesis-related pressures, and social disconnection as central sources of their psychological distress. The integration of both data sources provided a comprehensive understanding of the relationships identified through the statistical analyses and enriched the interpretation of the observed regression and correlation effects.

This study employed a mixed-methods approach to investigate academic stress and mental health among international students in Changsha, China. The findings indicate that academic pressure, language barriers, and social isolation are key risk factors contributing to psychological distress among international students (Ahmad & Shah, 2018; Qian & Yu, 2023). Furthermore, the results are consistent with the Job Demands–Resources (JD-R) Theory and the Transactional Model of Stress and Coping, suggesting that students experience elevated levels of stress when academic and acculturative demands exceed their available coping resources and the support provided by institutional systems (Ding, 2016; Cao & Meng, 2023; Raja et al., 2023).

While the quantitative results show an alarming number of mental health symptoms among the study population, they did show some positive news. Around 40% of students responded that they feel stressed either often or always. Anxiety/depressive symptoms were also experienced at a high rate (reaching 60% of responses for ‘stress inclusive’ measurement). This confirms previous research about the damages of these pressures on international students (Alam et al., 2021; Yuan et al., 2024).

The results indicated that academic workload had a significant role in predicting the three mental health measures (Levecque et al., 2017). Hierarchical regression analysis revealed that academic load was the heaviest predictor to all three mental health outcomes, and the standardized beta coefficients ranged from 0.31 to 0.37 for all three sets of regressions. All three variables had positive correlations with academic load, with moderate effects of 0.51–0.58. These results reaffirm previous findings on the negative influence of academic work overload on mental health among university students (B. Yu et al., 2014).

The qualitative results can help to explain these quantitative trends. Several students provided similar descriptive accounts of the nonstop assignment, exam, presentation and research cycle (Abbas et al., 2014). Postgraduate students described the research and publication requirements that posed particular challenges, since they are required to produce more than just a thesis (Troutman et al., 2022; Raksithaa, 2024). Nearly all the survey respondents described pressure to publish in elite journals in the fields of their institution and region, as well as across the nation, as a major academic stressor. The individual ranks of these stressors were summarized in previous table.

Degree-level differences were also determined from graduate student data, which showed that master’s students also had the highest percentage of high stress imagined situations (35.6%), followed by PhD students (28.6%), and undergraduates (25.0%). This may reflect that the transition from undergraduate to postgraduate studies is an academic process in itself; therefore, more growing indications of self-independent work and/or research productivity are expected. These graduate students, however, may have had certain coping mechanisms and support networks in place due to their academic experiences, making them more susceptible to the alleviating effects of such supports (Guan et al., 2023).

Beyond academic demands, social and cultural factors also played a significant role in shaping students’ mental health experiences. Social isolation and language barriers emerged as significant predictors of increased stress, anxiety, and depression across all mental health indicators (Jiang et al., 2018). Correlation analysis revealed moderate positive relationships between these two factors and all measures of mental health. Interviews revealed that for some students, language differences led to social withdrawal and a sense of alienation from their peers, and limited their capacity to build meaningful relationships (Ding, 2016). Many participants described a disconnect not only in the classroom but also in their extracurricular activities and daily life in general. A few students reported feeling that they did not belong and had trouble navigating different facets of their new environment. These findings support Berry’s acculturative stress framework, emphasizing the psychological burden associated with adapting to a new cultural context (Liu et al., 2024; Yuan et al., 2024).

While it is not possible to draw any conclusive statements on this study, there are several important lessons learned and implications from these findings that should be addressed and considered by those who work in this field (Dwumah Manu et al., 2023). Of particular interest to the current study is the use of institutional support services. Although they all visited the writing center and participated in the thesis/dissertation workshops, the contact hours and satisfaction levels were higher than those of counseling/psychological services (Cao & Meng, 2023). The qualitative interviews, however, revealed that some of the potential reasons for the utilization or non-utilization of institutional services are due to cultural stigma attached to seeking help for mental health issues and lack of linguistic access points (Demerouti & Bakker, 2023; Liu et al., 2024).

Qualitative findings further support the visibility of informal coping supports. Not surprisingly, stress management strategies were often dependent upon peer networks, friendships, exercise, religious activities and strength, and other informal sources of support. However, virtually all of these students interviewed stated that although these forms of coping emotional self-care were helpful, they were often insufficient in quelling relentless academic and social stressors (Dong et al., 2024). These findings are consistent with the JD-R perspective that personal resources are most effective when they are sustained by institutional and social resources.

Finally, these regression models only explained a modest amount of variance in mental health outcomes (R^2^ = 0.154–0.157). As such, there are likely other factors that also influence general health, mental health, and mental health-specific stigma originating from within the individual (e.g., personality traits, pre-existing mental health conditions, family support, and individual coping styles) and from one’s environment (e.g., discrimination, acculturative stress, and access to mental health resources). It is recommended that these variables be examined in future research to better understand the complex and dynamic nature of mental health in international students.

In conclusion, the integration of quantitative and qualitative data in this explanatory sequential design reveals convergent evidence: academic workload, social isolation, and language barriers are significant predictors of psychological distress, and the qualitative narratives clarify the mechanisms driving these associations. While quantitative findings establish the magnitude and significance of these relationships, qualitative data explain how these stressors manifest in students’ daily lives, why they are particularly distressing, and what barriers prevent students from seeking support. This integrated understanding underscores the need for comprehensive, culturally responsive interventions that address both academic and social dimensions of international students’ experiences.

Addressing these challenges is important for Chinese universities to do well with higher education globalization as this supports students’ mental health, academic achievement and retention, and international students’ educational process.

## 5. Conclusions

The findings indicate that international students in Changsha experience substantial academic demands alongside the challenges associated with cultural adaptation. Evidence from both the qualitative and quantitative components of the study identifies academic stress—particularly pressures related to research and publication requirements, thesis and dissertation completion, and heavy coursework—as a primary contributor to psychological distress (Mao et al., 2019). These academic pressures are further intensified by language barriers and social isolation, while institutional support services remain unevenly available and are often underutilized due to cultural and contextual constraints. Master’s students were noted to be the most stressed, indicating that this would be one population to focus on. The level of stress experienced by this age group is a significant finding, as it reflects the academic transition from structured undergraduate coursework to the greater independence and self-directed learning required in postgraduate research. In addition, the study highlights dissonance of students’ psychological needs with provision at institutions of higher education, which indicates areas for further institutional support. Finally, the study provides a comparison study of international students’ wellbeing, and the unique way that global educational pressures can manifest themselves within specific local educational regimes and cultural contexts.

In conclusion, when the university in Changsha accommodates the large distribution of international students over some time, it may acknowledge the crisis that even though they are mature students, their various needs are not being well accommodated with an appropriate support system. Closing these gaps with improved culturally sensitive interventions and support systems for students’ studies, or counseling, academic advising and mentoring resources, language support and active social integration initiatives should be prioritized as they could promote resilience, mental health and educational success for international students studying in China.

### Study Limitations

There are still some limitations of this study. They should be taken into account when addressing the results. The cross-sectional design does not allow for causal conclusions. Self-reported measures may have failed to get through to the actual demographic groups, which could make the response biased. Social desirability, attitude towards mental ill health, and mental health stigma can all have an impact on responses as well. The in-depth qualitative interviews are not able to draw a representative sample of all college students’ experiences of being international students, but instead provide nuanced experiences of the selected sample. Some researchers have argued that the small sample in qualitative interviews could lead to generalizations of limited participants to the whole student population in a university. The sample of this study was somewhat limited: only 10 students, all from Africa and Asian background types, in four Changsha-based Chinese universities. The findings from this sample may not be similar throughout the entirety of China. This could be because of cultural differences. Future studies should be conducted using a larger sample for more representative findings in various cultural settings.

This study has several limitations. The quantitative sample was drawn from only four universities in Changsha, and the qualitative component included 10 international students, which may limit the generalizability of the findings to other regions of China with different institutional and cultural contexts. In addition, the absence of a formal nonresponse analysis and the modest explanatory power of the regression models (R^2^ = 0.152–0.157) suggest that other important factors, such as psychological resources, pre-arrival mental health, family support, and coping strategies, should be examined in future research. Furthermore, mental health outcomes were measured using brief self-reports rather than standardized psychometric instruments, such as the DASS-21 or PHQ-9, which may have reduced the precision of symptom assessment.

## Figures and Tables

**Table 1:** Confirmatory Factor Analysis (CFA) Model Fit Indices (N = 500).

Model	χ^2^ (df)	*p*-Value	CFI	TLI	RMSEA (90% CI)	SRMR
Overall Measurement Model	412.85 (224)	<0.001	0.93	0.92	0.052 (0.045–0.058)	0.047

**Table 2:** Demographic Characteristics of international students in Changsha (N = 500).

Variable	Category	Frequency (n)	Percentage (%)
Gender	Male	280	56.0
	Female	220	44.0
Age	20–25	134	26.8
	26–30	197	39.4
	31–40	169	33.8
Region	Africa	210	42.0
	Asia	290	58.0
Degree Level	Undergraduate	100	20.0
	Master’s	225	45.0
	PhD	175	35.0

**Table 3:** Prevalence of mental health symptoms.

Symptom	Never (%)	Sometimes (%)	Often (%)	Always (%)
Stress	12.0	48.0	32.0	8.0
Anxiety	18.0	42.0	28.0	12.0
Depression	22.0	38.0	27.0	13.0
Sleep disturbance	25.0	40.0	25.0	10.0
Fatigue/burnout	20.0	45.0	25.0	10.0

Note: Stress, anxiety, and depression were measured using 4-point Likert-type frequency scales. “Quite a lot” and “A lot” were categorized as moderate to high levels for analytical purposes, following established survey research conventions in student mental health studies.

**Table 4:** Hierarchical regression analyses predicting stress, anxiety, and depression among international students in Changsha (N = 500).

Predictor	Stress β [95% CI] (FDR-adj *p*)	Anxiety β [95% CI] (FDR-adj *p*)	Depression β [95% CI] (FDR-adj *p*)
Step 1: Demographics			
Female (ref: Male)	0.18 [0.04, 0.32] (0.015)	0.22 [0.08, 0.36] (0.008)	0.20 [0.06, 0.34] (0.011)
Age	−0.07 [−0.15, 0.01] (0.096)	−0.05 [−0.13, 0.03] (0.148)	−0.08 [−0.16, 0.00] (0.086)
Step 1 R^2^/ΔR^2^	0.017/0.017	0.021/0.021	0.019/0.019
Step 2: Academic Factors			
Master’s	0.16 [0.08, 0.40] (0.090)	0.14 [0.06, 0.38] (0.112)	0.12 [0.04, 0.36] (0.136)
PhD	0.24 [−0.02, 0.34] (0.009)	0.26 [−0.01, 0.36] (0.007)	0.28 [0.01, 0.38] (0.005)
Academic Load	0.37 [0.25, 0.49] (<0.001)	0.34 [0.22, 0.46] (0.001)	0.31 [0.19, 0.43] (0.003)
Step 2 R^2^/ΔR^2^	0.119/0.102	0.128/0.107	0.122/0.103
Step 3: Social Factors			
Social Isolation	0.22 [0.08, 0.36] (0.006)	0.19 [0.05, 0.33] (0.015)	0.25 [0.11, 0.39] (0.004)
Language Barrier	0.15 [0.03, 0.27] (0.020)	0.17 [0.05, 0.29] (0.012)	0.14 [0.02, 0.26] (0.024)
Step 3 R^2^/ΔR^2^	0.154/0.035	0.152/0.024	0.157/0.035
Total Model R^2^	0.154	0.152	0.157

Note: All regression models were tested for multicollinearity (VIF < 5) and robustness across alternative specifications.

**Table 5:** Joint display: Quantitative findings and supporting qualitative themes.

Quantitative Finding	Qualitative Theme	Illustrative Quote
Academic load—strongest predictor of stress (β = 0.37, *p* < 0.001)	Overwhelming and cumulative workload	“When I complete one task, several more appear. It’s a cycle that never ends.”
Research/publication burden—highest mean stressor (M = 4.50)	Pressure from research and publication	“My supervisor insisted I withdraw from a good Q2 journal to target only Q1. It doubled my stress.”
Social isolation—significant predictor of depression (β = 0.25, *p* < 0.01)	Cultural distance and loneliness	“After six months, I still eat alone most days… I feel disconnected from people around me.”
Language barriers—significant predictor of anxiety (β = 0.17, *p* < 0.05)	Language as social and academic barrier	“Even after months here, I still struggle to communicate effectively in class and outside.”
Low counseling utilization (38%) and satisfaction (M = 3.02)	Cultural stigma and limited awareness	“In my culture, seeking psychological help feels shameful. Even if services exist, I hesitate to use them.”

**Table 6:** Correlation matrix of stressors and mental health (N = 500).

Variable	1	2	3	4	5	6
1. Academic Load	1.00					
2. Language Barrier	0.43 **	1.00				
3. Social Isolation	0.31 **	0.34 **	1.00			
4. Stress	0.58 **	0.42 **	0.27 **	1.00		
5. Anxiety	0.53 **	0.39 **	0.29 **	0.68 **	1.00	
6. Depression	0.51 **	0.36 **	0.32 **	0.65 **	0.74 **	1.00

Note: ** *p* < 0.01 (FDR-corrected). All values are Pearson’s correlation coefficients (r).

**Table 7:** Validity and reliability of study instruments.

Construct	No. of Items	Cronbach’s Alpha	CR	AVE	CFA Loading Range
Academic Stress	7	0.81	0.82	0.45	0.53–0.79
Anxiety	7	0.84	0.84	0.51	0.56–0.78
Depression	7	0.74	0.80	0.53	0.51–0.75
Support Services	3	0.73	0.73	0.40	0.45–0.72
Language Barriers	7	0.76	0.78	0.48	0.55–0.77
Social Isolation	7	0.79	0.80	0.49	0.52–0.76

**Table 8:** Stress level distribution by degree level among international students in Changsha (N = 500).

Degree Level	Low Stress n (%)	Moderate Stress n (%)	High Stress n (%)	Total
Undergraduate	22 (22.0%)	53 (53.0%)	25 (25.0%)	100
Master’s	37 (16.4%)	108 (48.0%)	80 (35.6%)	225
PhD	45 (25.7%)	80 (45.7%)	50 (28.6%)	175
Total	104 (20.8%)	241 (48.2%)	155 (31.0%)	500

**Table 9:** Top academic stressors among international students (Mean scores, 1–5 scale).

Rank	Stressor	Mean (SD)
1	Research/Publication burden	4.50 (0.70)
2	Thesis/Dissertation pressure	4.10 (0.80)
3	Heavy academic workload	3.80 (0.90)
4	Examination pressure	3.60 (0.80)
5	Language barriers	3.10 (0.70)
6	Social isolation (new)	3.05 (0.75)
7	Financial stress (new)	2.95 (0.78)
8	Supervisor student relationship challenges (new)	2.85 (0.72)

**Table 10:** Utilization and satisfaction with institutional support services among international students (N = 500).

Support Service	Mean Satisfaction (SD)	Students Using Service n (%)
Counseling/psychological services	3.02 (1.28)	190 (38.0%)
Academic advising services	3.58 (0.82)	290 (58.0%)
Thesis/dissertation support workshops	4.05 (0.74)	325 (65.0%)
Language support/writing center assistance	3.40 (0.90)	240 (48.0%)
Peer mentoring/international student buddy programs	3.25 (0.88)	210 (42.0%)
Orientation & integration programs	3.70 (0.77)	310 (62.0%)

**Table 11:** The lived experience of international graduate student distress and resilience.

Theme	Sub Themes	Participant Context/Quotes
Academic Stress	Overwhelming and cumulative workload	“When I complete one task, several more appear. It’s a cycle that never ends.”
	Pressure from research and publication	“My supervisor insisted I withdraw from a good Q2 journal to target only Q1. It doubled my stress and delayed everything.”
	Heavy coursework and long classes	“The long-lasting classes of about 4 h, heavy workload including reading articles for assignments and presentations increases the academic load.”
Acculturative and Institutional Pressure	Language as a social barrier	“Language is one of the biggest challenges. Even after months here, I still struggle to communicate effectively in class and outside.”
	Cultural distance and loneliness	“Back home, my classmates and I would study together daily and share meals. Here, after six months, I still eat alone most days.”
	Logistical challenges	“Waking up early in the morning, waiting for buses to move to another campus has affected my mental health.”
Mental Health Distress	Emotional and physical depletion	“I am always tired. Even when I sleep, I’m thinking about my paper. Headaches are normal now. Sometimes I just feel completely empty.”
	Anxiety and stress symptoms	“The pressure of deadlines, presentations, and research makes me constantly anxious and fatigued.”
Coping: Between Resilience and Exhaustion	Reliance on peer networks	“My only saving grace is my group of friends from my country. We vent, we cook together, we understand each other without explaining.”
	Self-care and coping strategies	“I try to exercise and meditate, but it is not enough to counter the stress from research and classes.”
Supervisor Student Interaction Stress	Conflicting expectations	“I wrote the paper targeting a specific journal, but my supervisor insisted on a higher-impact journal. Balancing my goals with their expectations is exhausting.”
	Lack of guidance or over involvement	“Sometimes the supervisor’s interference increases stress rather than helping me; I feel caught between their instructions and my research plan.”
Institutional Support Gaps	Limited awareness or accessibility of services	“Even though counseling is available, I don’t know how to access it, and language is a barrier in communicating with staff.”
	Cultural stigma around mental health	“In my culture, seeking psychological help feels shameful. Even if services exist, I hesitate to use them.”

## Data Availability

The authors declare that the data supporting the findings of this study are available from the corresponding author upon reasonable request. The data are not publicly available due to ethical and confidentiality considerations. Contact information of the corresponding author is asimzubairmalik@gmail.com.

## References

[B1-behavsci-16-01212] Abbas M. Z., Liang X.-M., Liu K.-Z. (2014). An evaluation of acculturation stress, mental health issues and adaptation difficulties of international students in China. Sichuan Mental Health.

[B2-behavsci-16-01212] Ahmad A. B., Shah M. (2018). International students’ choice to study in China: An exploratory study. Tertiary Education and Management.

[B3-behavsci-16-01212] Alam M. D., Lu J., Ni L., Hu S., Xu Y. (2021). Psychological outcomes and associated factors among the international students living in China during the COVID-19 pandemic. Frontiers in Psychiatry.

[B4-behavsci-16-01212] An R., Chiang S. Y. (2015). International students’ culture learning and cultural adaptation in China. Journal of Multilingual and Multicultural Development.

[B5-behavsci-16-01212] Bagozzi R. P., Yi Y. (1989). The degree of intention formation as a moderator of the attitude-behavior relationship. Social Psychology Quarterly.

[B6-behavsci-16-01212] Bakker A. B., Demerouti E. (2017). Job demands–resources theory: Taking stock and looking forward. Journal of Occupational Health Psychology.

[B7-behavsci-16-01212] Berry J. W. (2006). Contexts of acculturation. The Cambridge handbook of acculturation psychology.

[B8-behavsci-16-01212] Braun V., Clarke V. (2006). Using thematic analysis in psychology. Qualitative Research in Psychology.

[B9-behavsci-16-01212] Cao C., Meng Q. (2023). The dual processes of health impairment and motivation in international student adjustment in China: Insights from a demands-resources model. Current Psychology.

[B10-behavsci-16-01212] Cao C., Zhang J., Meng Q. (2023). A social cognitive model predicting international students’ cross-cultural adjustment in China. Current Psychology.

[B11-behavsci-16-01212] Creswell J. W. (1999). Mixed-method research: Introduction and application. Handbook of educational policy.

[B12-behavsci-16-01212] Demerouti E., Bakker A. B. (2023). Job demands-resources theory in times of crises: New propositions. Organizational Psychology Review.

[B13-behavsci-16-01212] Ding X. (2016). Exploring the experiences of international students in China. Journal of Studies in International Education.

[B14-behavsci-16-01212] Dong W., Tang H., Wu S., Lu G., Shang Y., Chen C. (2024). The effect of social anxiety on teenagers’ internet addiction: The mediating role of loneliness and coping styles. BMC Psychiatry.

[B15-behavsci-16-01212] Dwumah Manu B., Ying F., Oduro D., Antwi J., Yakubu Adjuik R. (2023). The impact of social media use on student engagement and acculturative stress among international students in China. PLoS ONE.

[B16-behavsci-16-01212] Eisenberg D., Gollust S. E., Golberstein E., Hefner J. L. (2007). Prevalence and correlates of depression, anxiety, and suicidality among university students. American Journal of Orthopsychiatry.

[B17-behavsci-16-01212] Fornell C., Larcker D. F. (1981). Evaluating structural equation models with unobservable variables and measurement error. Journal of Marketing Research.

[B18-behavsci-16-01212] Gong Y., Gao X., Li M., Lai C. (2021). Cultural adaptation challenges and strategies during study abroad: New Zealand students in China. Language, Culture and Curriculum.

[B19-behavsci-16-01212] Guan M., Fan X., Li J. (2023). Pedagogical doctoral students in China under pressure: An empirical analysis of CSSCI journals. Problems of Education in the 21st Century.

[B20-behavsci-16-01212] Hair J. F., Risher J. J., Sarstedt M., Ringle C. M. (2019). When to use and how to report the results of PLS-SEM. European Business Review.

[B21-behavsci-16-01212] Hussain M., Shen H. (2019). A study on academic adaptation of international students in China. Higher Education Studies.

[B22-behavsci-16-01212] Ivankova N. V., Creswell J. W., Stick S. L. (2006). Using mixed-methods sequential explanatory design: From theory to practice. Field Methods.

[B23-behavsci-16-01212] Jiang Q., Li Y., Shypenka V. (2018). Loneliness, individualism, and smartphone addiction among international students in China. Cyberpsychology, Behavior, and Social Networking.

[B24-behavsci-16-01212] Levecque K., Anseel F., De Beuckelaer A., Van der Heyden J., Gisle L. (2017). Work organization and mental health problems in PhD students. Research Policy.

[B25-behavsci-16-01212] Li X. (2015). International students in China: Cross-cultural interaction, integration, and identity construction. Journal of Language, Identity & Education.

[B26-behavsci-16-01212] Liu S., Wei M., Ko S., Du Y., Wang C., He L., Tsai P. C. (2024). Engagement with new possibilities, personal growth initiative, and optimism among East Asian international students. Journal of Asia Pacific Counseling.

[B27-behavsci-16-01212] Ma J., Zhao K. (2018). International student education in China: Characteristics, challenges, and future trends. Higher Education.

[B28-behavsci-16-01212] Mao Y., Zhang N., Liu J., Zhu B., He R., Wang X. (2019). A systematic review of depression and anxiety in medical students in China. BMC Medical Education.

[B29-behavsci-16-01212] Qian J., Yu J. (2023). Effects of Chinese language learning anxiety on the mental health of international students in China: The chain mediating effect of campus adaptation and academic resilience. Psychology Research and Behavior Management.

[B30-behavsci-16-01212] Raja R., Ma J., Zhang M., Li X. Y., Almutairi N. S., Almutairi A. H. (2023). Social identity loss and reverse culture shock: Experiences of international students in China during the COVID-19 pandemic. Frontiers in Psychology.

[B31-behavsci-16-01212] Raksithaa S. (2024). Publication stress amongst scholars and faculties: A concern of mental health. Mental Health and Social Inclusion.

[B32-behavsci-16-01212] Sun J., Dunne M. P., Hou X. Y. (2012). Academic stress among adolescents in China. Australasian Epidemiologist.

[B33-behavsci-16-01212] Troutman J., Riley D., Mallouk K. (2022). Visualizing stress and relief: How stressors and coping mechanisms interact in engineering graduate student experiences. 2022 ASEE Annual Conference & Exposition.

[B34-behavsci-16-01212] Wang S., Li H., Chen X., Yan N., Wen D. (2023). The mediating role of psychological capital in the association between life satisfaction and depressive and anxiety symptoms among Chinese medical students during the COVID-19 pandemic: A cross-sectional study. BMC Psychiatry.

[B35-behavsci-16-01212] Xu X., Li X. M., Zhang J., Wang W. (2018). Mental health-related stigma in China. Issues in Mental Health Nursing.

[B36-behavsci-16-01212] Yang Z., de Wit H. (2019). International students in China: Facts, paths, and challenges. International Higher Education.

[B37-behavsci-16-01212] Yu B., Chen X., Li S., Liu Y., Jacques-Tiura A. J., Yan H. (2014). Acculturative stress and influential factors among international students in China: A structural dynamic perspective. PLoS ONE.

[B38-behavsci-16-01212] Yu S., Kowitt S. D., Fisher E. B., Li G. (2018). Mental health in China: Stigma, family obligations, and the potential of peer support. Community Mental Health Journal.

[B39-behavsci-16-01212] Yuan Q., Lu X., Shi X., Leng J., Fan Z. (2024). Impact of the sense of power on loneliness among international students in China: The chain mediating role of perceived discrimination and loneliness stigma. BMC Psychology.

[B40-behavsci-16-01212] Zhu J., Gu M., Yang L., Xun S., Wan M., Li J. (2022). Academic adaptation of international students in China: Evidence from the grounded theory and structure equation model. Sustainability.

